# Findings on In Vitro Transporter-Mediated Drug Interactions and Their Follow-Up Actions for Labeling: Analysis of Drugs Approved by US FDA between 2017 and 2021

**DOI:** 10.3390/pharmaceutics14102078

**Published:** 2022-09-29

**Authors:** Kyeong-Ryoon Lee, Ji-Eun Chang, Jongmin Yoon, Hyojeong Jin, Yoon-Jee Chae

**Affiliations:** 1 Laboratory Animal Resource Center, Korea Research Institute of Bioscience and Biotechnology, Cheongju 28116, Korea; 2 Department of Bioscience, University of Science and Technology, Daejeon 34113, Korea; 3 College of Pharmacy, Dongduk Women’s University, Seoul 02748, Korea; 4 College of Pharmacy, Woosuk University, Wanju 55338, Korea; 5 Research Institute of Pharmaceutical Sciences, Woosuk University, Wanju 55338, Korea

**Keywords:** drug interactions, drug transporters, inhibitors, substrates

## Abstract

Understanding possible follow-up actions on in vitro findings helps determine the necessity of labeling for drug interactions. We analyzed information for in vitro findings on transporter-mediated interactions of drugs approved by the U.S. Food and Drug Administration’s Center for Drug Evaluation and Research for the last five years (i.e., 2017–2021) and their follow-up actions for labeling. Higher R values than the pre-defined cut-off were observed with 3.7–39.1% inhibitor drugs in a simple prediction. Among these drugs, 16–41.7% were labeled with their potential drug interactions, while results of supporting studies or scientific rationales were submitted for the other drugs leading to no interaction labeling. In vitro transporter substrates were reported with 1.7–67.6% of drugs. The interaction labels for these substrate drugs were observed in up to 40% of drugs, while the other drugs were not labeled on the drug interactions with claims for their low interaction potential, evidenced by clinical studies or scientific rationales. The systematic and comprehensive analysis in this study will provide insight into the management of in vitro findings for transporter substrate or inhibitor drugs.

## 1. Introduction

The maintenance of drug concentrations within the therapeutic range is crucial for achieving tolerable and effective treatment in patients. Drug concentrations depend on patient factors such as age, disease stage, lifestyle, genetic factors, diet, and the function of tissues governing drug elimination, including the liver or kidney, and co-administered drugs [1]. Concomitant medications often inhibit drug-metabolizing enzymes or transporters, altering drug kinetics and leading to changes in drug concentrations within the body. Such unanticipated alterations in drug concentration may induce treatment failure and increase the risk of adverse events. An earlier study showed that 29.9% of adverse drug reactions directly related to hospital admissions were presumably due to drug interactions [2]. Similarly, drug interactions accounted for 33.1% of preventable adverse drug reactions [3], indicating that drug interactions should be fully investigated to protect patients from avoidable risks. 

Drug transporters are one of the determinants of drug pharmacokinetics [4]. More than 400 membrane transporters have been identified and classified into two major subfamilies: ATP-binding cassettes (ABC) and solute carriers (SLC) [5]. P-glycoprotein (P-gp) and breast cancer resistance protein (BCRP) are ABC transporters frequently involved in drug interactions [6,7]. They are expressed in several organs, including the intestine, liver, and kidney, restricting the absorption of drugs or facilitating their excretion [8,9]. Organic anion transporting polypeptide (OATP) 1B1 and OATP1B3 transporters are expressed in the liver and govern drug transport into the liver [10]. Organic anion transporter (OAT) 1, OAT3, and organic cation transporter 2 (OCT2) are expressed in the basolateral membrane of the renal proximal tubule [11]. Multidrug and toxin extrusion (MATE) 1 and MATE2-K proteins, expressed on the brush border membrane, play a role in the active renal secretion of drugs in the kidney [12]. These nine transporters (P-gp, BCRP, OATP1B1, OATP1B3, OAT1, OAT3, OCT2, MATE1, and MATE2-K) are primarily associated with drug interactions, and the co-administration of inhibitor drugs for transporters with substrate drugs may induce serious adverse events or treatment failure [13,14].

Considering the significant roles of drug interactions in disease treatment, the potential of drug interactions should be thoroughly evaluated. Over the years, various in vitro methodologies have been developed to evaluate transporter-mediated drug interactions [15,16]. However, in vitro findings alone are not enough to predict clinically significant drug interactions. Drug interaction studies in humans or translation of in vitro findings to clinical practice can provide critical information on clinical drug interactions that are finally reflected in drug labels [17,18]. In line with this, the Food and Drug Administration (FDA) of the United States (US) provides relevant guidelines representing the agency’s current opinion on drug interaction studies [19,20]. While these guidelines are not mandatory for drug developers, they are usually considered the gold standard for evaluating drug interactions. Guidance for in vitro drug interaction studies provides information on how in vitro results can outline future clinical drug interaction studies [19]. For example, a basic method can be applied to calculate R values, which is an indicator to determine whether to consider proceeding with clinical drug interaction studies. However, clinical studies are not mandatory for all drugs with R values higher than the pre-defined cut-off values. If drug interactions are not expected to be significant by scientifically reasonable rationales based on physicochemical properties (e.g., permeability and solubility), elimination route, and treatment regimens, or if the physiologically based pharmacokinetic modeling (PBPK) approach estimates negligible interactions, further clinical studies may be exempted with no labeling of drug interactions. This aids in reducing unnecessary clinical studies in humans and cost-effective drug development. 

Thus, we have analyzed the follow-up actions for in vitro transporter substrate or inhibitor drugs approved by the FDA’s Center for Drug Evaluation and Research (CDER) over the last five years (2017–2021). First, we collected information on in vitro findings from the new drug application (NDA) approval packages. For in vitro transporter inhibitors, we calculated the R values using a basic method to estimate the necessity of clinical drug interaction studies. Information on in vitro substrates was also collected from the FDA review documents. We then analyzed the drug labels according to the statement for drug interactions. For drugs with labels about interactions, information on supporting evidence for labeling was analyzed. In the case of drugs without labeling on drug interactions, despite higher R values than the cut-off, information on scientific rationales or other follow-up actions was investigated. This systematic and comprehensive analysis will provide insight into the management of in vitro findings for transporter substrate or inhibitor drugs.

## 2. Materials and Methods

### 2.1. Data Collection

The drug list approved by the FDA’s CDER for the last five years (i.e., 2017–2021) was obtained from the FDA website (https://www.fda.gov/drugs/development-approval-process-drugs/new-drugs-fda-cders-new-molecular-entities-and-new-therapeutic-biological-products; accessed on 10 January 2022). Biologic drugs, siRNA, oligonucleotides, peptides, diagnostic agents, and topically used drugs were excluded from the analysis. 

FDA review documents for NDA were extracted from the online database of FDA-Approved Drugs (https://www.accessdata.fda.gov/scripts/cder/daf/index.cfm), and information on P-gp, BCRP, OATP1B1, OATP1B3, OAT1, OAT3, OCT2, MATE1, and MATE2-K was obtained from the documents. For the analysis of transporter inhibitors, the half-maximal inhibitory concentration (IC_50_) or inhibition constant (K_i_) values of drugs on each transporter (obtained from the in vitro study), maximum plasma concentration (C_max_), unbound fraction of drugs in plasma (f_u,p_), absorption rate constant (k_a_), fraction absorbed (F_a_), and intestinal availability (F_g_) of drugs were collected from FDA review documents (e.g., clinical pharmacology and biopharmaceutics reviews, non-clinical reviews, and multidiscipline reviews). For some drugs, IC_50_ values were not available owing to limitations in increasing test concentrations. In these cases, as a conservative approach, the highest concentrations were assumed to be IC_50_ values if the inhibition percentage was more than 20% at the highest concentration tested. Information on the dosing route, recommended maximum dosage, and any update on information for drug interactions (compared to the timing of drug approval) was obtained from the latest version of drug labels available on the FDA website. For the analysis of transporter substrates, the results from an in vitro study, aiming to determine whether the drugs are substrates of transporters, were collected from FDA review documents. All information was collected for parent drugs and their metabolites, based on their availability. For combination drugs, information on each component was collected. 

### 2.2. Application of the Basic Method for In Vitro Transporter Inhibitors

To determine candidates for drug labeling on transporter inhibition-mediated interactions, a basic method was used for in vitro transporter inhibitors according to the latest version of the FDA guidelines [19]. R values were calculated not only for parent drugs but also for metabolites if the information was available. When the calculated R values are higher than or equal to the pre-defined values, it was concluded that follow-up actions, such as clinical drug interaction studies, should be considered.

For P-gp and BCRP, R values were calculated using the equation below for orally administered drugs.

R = I_gut_ /IC_50_ or K_i_

where I_gut_ is the concentration of inhibitors in intestinal fluid, calculated by dividing the recommended maximum dose of the inhibitor by intestinal fluid volume in humans (250 mL), IC_50_ is the half-maximal inhibitory concentration, and K_i_ represents the inhibition constant. The pre-defined cut-off value for R was 10 [19,21].

For drugs administered by parenteral route or for metabolites, R values were calculated using the following equation:

R = I_1_/IC_50_ or K_i_

where I_1_ is the C_max_ of the drugs administered via parenteral route or metabolites. The pre-defined cut-off value was set at 0.1.

For OATP1B1 and OATP1B3, R values were obtained using the following equation [22,23]:

R = (f_u,p_ × I_in,max_)/IC_50_

Here, f_u,p_ is the unbound fraction of drugs in plasma, and I_in,max_ is the estimated maximum inhibitor concentration in plasma, at the inlet to the liver, and is calculated as follows:

I_in,max_ = I_max_ + (F_a_ × F_g_ × k_a_ × Dose)/Q_h_/R_B_

where I_max_ is the C_max_ of the inhibitor drug, F_a_ is the fraction absorbed, F_g_ is the intestinal availability, k_a_ is the absorption rate constant, Q_h_ is the hepatic blood flow rate (assumed to be 1500 mL/min in a 70 kg subject) [24], and R_B_ is the blood-to-plasma concentration ratio. When F_a_, F_g_, or k_a_ values are not available, the worst-case scenario was assumed, which was that the drugs were absorbed rapidly with complete absorption, and F_a_, F_g_, and k_a_ were set to 1, 1, and 0.1/min, respectively. If the reported f_u,p_ value was less than 1%, the f_u,p_ of the drugs was set at 1%, considering the variability and uncertainties of the protein binding assay. When the calculated R values are greater than or equal to 0.1, it was concluded that follow-up actions such as clinical drug interaction studies should be considered.

For OAT1, OAT3, OCT2, MATE1, and MATE2-K, R values were calculated using the following equation [25]:

R = I_max,u_/IC_50_ or K_i_

where I_max,u_ represents the unbound C_max_ of the inhibitors, calculated by multiplying f_u,p_ by C_max_, when not reported. The pre-defined cut-off value was 0.1.

### 2.3. Analysis of Follow-Up Actions on Possible Clinical Inhibitors or Substrates of Transporters

For drugs with higher R values than pre-defined cut-off values in a simple prediction by the basic method, information on follow-up actions was analyzed. Due to the overlapping properties between transporters, follow-up actions were categorized for each subfamily of transporters. Using FDA review documents and approval letters, we investigated whether clinical interaction studies or PBPK modeling were performed to evaluate the potential of clinical drug interactions or whether the post-marketing requirement (PMR) on a further drug interaction study was issued. Drug labels were reviewed to determine whether warnings or precautions were presented for transporter-mediated drug interactions. When there was no statement on transporter-mediated drug interactions on labels, even though no further actions, such as clinical drug interaction study or PBPK modeling, were performed, we attempted to find further information on the scientific rationale for the absence of labeling. The collected information was categorized based on the statement on transporter-mediated drug interactions in the label and then subcategorized further based on follow-up actions or scientific rationales. For the case of transporter inhibitors, the categorized information was matched with R values calculated using the basic method for each transporter.

## 3. Results

### 3.1. General Findings

**Figure 1:**
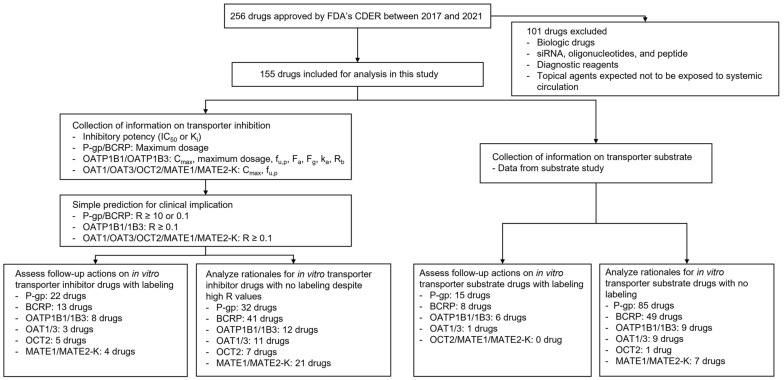
Workflow for the analysis using the drugs approved by FDA’s CDER between 2017 and 2021. F_a_, fraction absorbed; F_g_, intestinal availability; f_u,p_, unbound fraction of drugs in plasma; IC_50_, half maximal inhibitory concentration; k_a_, absorption rate constant; K_i_, inhibition constant, C_max_, maximum plasma concentration; R_b_, blood-to-plasma concentration ratio.

The overall study workflow and summary are shown in Figure 1. In total, 256 drugs were approved by the FDA’s CDER between 2017 and 2021 (Table 1). Excluding biological products, siRNAs, oligonucleotides, peptides, diagnostic agents, and topically used drugs, further analysis was performed on 155 drugs to evaluate follow-up actions for in vitro findings on transporter-mediated interactions. The list of drugs involved in further analysis and detailed information on the drugs are provided in Supplemental Table S1.

### 3.2. In Vitro Inhibitors and Their Follow-Up Actions

Among the 155 drugs involved in further analysis, information on P-gp, BCRP, OATP1B1, OATP1B3, OAT1, OAT3, OCT2, MATE1, and MATE2-K inhibition was available for 144, 138, 137, 135, 135, 133, 137, 108, and 104 drugs, respectively (Table 2 and Supplemental Table S2). More than 80% of the drugs were evaluated every year for their inhibitory effects on P-gp, BCRP, OATP1B1, and OATP1B3. Information on the inhibitory effects of drugs on OAT1, OAT3, and OCT2 was available for approximately 80% of drugs. In contrast, only 25% of the total drugs were evaluated for inhibitory effects on MATE1 and MATE2-K in 2017 and after then the study of the transporters increased rapidly every year. On average, 69.7 and 67.1% of the drugs approved between 2017 and 2021 had information on MATE1 and MATE2-K inhibition, respectively. For the last three years (2019–2021), information on the inhibitory effects on all transporters was available for more than 80% of drugs in FDA review documents, indicating the critical role of evaluation of transporter inhibition in drug development in recent years.

In this study, we focused on the potential clinical implications of the inhibitory effects based on the basic method calculating the R values. As a result, the R values of 37.5 and 39.1% of drugs or their metabolites for P-gp and BCRP, respectively, were higher than the cut-off values. In contrast, less than 20% of the drugs had higher R values than the cut-off for the other transporters, demonstrating a broader specificity of P-gp and BCRP for drugs (Table 2).

**Figure 2:**
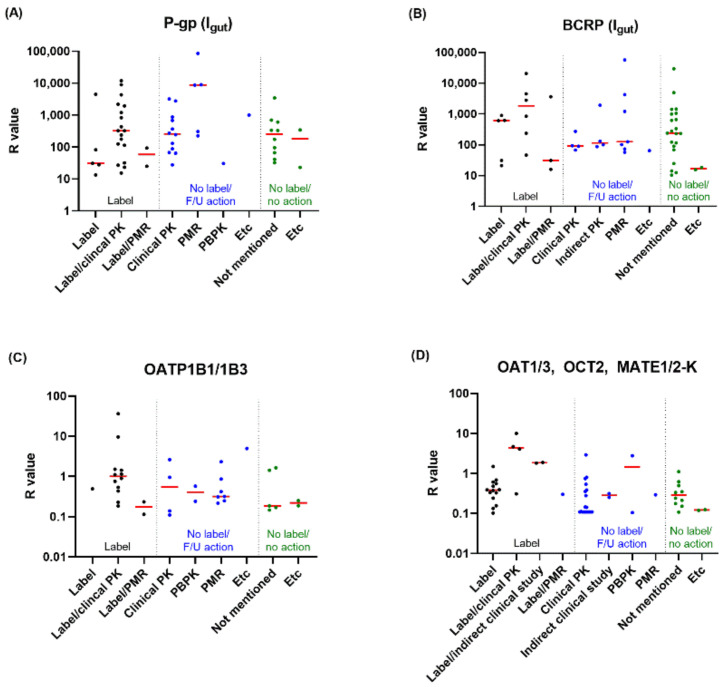
R values of *in vitro* inhibitor drugs and their follow-up actions (only for drugs with calculated R ≥ cut-off value) for P-gp (**A**); based on I_gut_), BCRP (**B**); based on I_gut_), OATP1B1/1B3 (**C**), and OAT1/3, OCT2, and MATE1/2-K (**D**). Red lines represent the median values of R in each group. F/U, follow-up; I_gut_, the concentration of inhibitors in intestinal fluid; PK, pharmacokinetics; PBPK, physiologically based pharmacokinetics; PMR, post-marketing requirement.

A further analysis was performed for drugs with R values higher than the cut-off to investigate the follow-up actions for the transporter inhibition observed in vitro (Table 3, Figure 2, Supplemental Table S3). Twenty-two drugs contained a statement on drug interactions by P-gp inhibition in label. Among these drugs, 17 were labeled based on observations in a clinical drug interaction study. A PMR was issued for two drugs to perform a clinical drug interaction study to elucidate the pharmacokinetic impact of P-gp-mediated interactions, while labels describe potential drug interactions in advance. Among the 32 drugs with no label on P-gp inhibition-mediated drug interactions, 13 were claimed for their low potential for drug interactions by a clinical drug interaction study with a P-gp specific substrate, and concern of drug interactions for two other drugs was resolved by PBPK modeling. The PMR was issued for five drugs to perform a clinical interaction study with a P-gp substrate, which was not mentioned on the label. Low solubility and low potential for transporter–drug contact were other reasons for the absence of statements on P-gp drug interactions on labels. No further actions or rationales to claim low drug interaction potential were found for 10 drugs in FDA review documents or labels.

Statements on BCRP inhibition-mediated interactions were accessible on the labels of 13 drugs. Clinical drug interaction studies for six drugs showed significant drug interactions with BCRP substrates, leading to a corresponding statement in the labels. The PMR was issued for three drugs to investigate drug interactions for BCRP in humans, and potential drug interactions were mentioned on the label in advance. No further action was reported for the four drugs, although their labels indicated the possibility of BCRP inhibition-mediated drug interactions. The labels of 41 drugs with high R values did not present potential drug interactions via BCRP inhibition. Clinical drug interaction studies showed no relevant interactions for five drugs, and PMR was issued for seven drugs to perform clinical drug interaction studies or PBPK modeling to clarify the potential of the interactions by BCRP inhibition. The claims of low potential for BCRP inhibition-mediated interactions for four drugs were justified by comparing IC_50_ values of BCRP and P-gp and clinical interaction study results for P-gp (Table 3; tepotinib, lefamulin, betrixaban, and telotristat ethyl; classified as an indirect clinical study in no label category). Similar to P-gp, low solubility and no drug–transporter contact were suggested as reasons for not labeling drug interactions for BCRP. However, 40.7% of drugs were not described for their potential for BCRP inhibition-mediated drug interactions on the label, although no further actions were presented or scientific rationales were suggested. Among them (a total of 22 drugs), 17 drugs are anti-cancer agents and there may be some different approaches with regard to labeling between cancer and non-cancer agents even though FDA guidance does not specifically differentiate the risk management of drug interactions depending on indications. We analyzed follow-up actions regardless of indications in this study, and investigations on this depending on indications would provide insight for management of in vitro findings on drug interactions in specific diseases such as cancer. For the hepatic transporters OATP1B1 and OATP1B3, eight drugs were labeled with potential drug interactions via OATP inhibition. Among the nine drugs investigated for their potential for clinical drug interactions in humans, six had the potential to induce significant drug interactions via OATP inhibition, whereas three drugs (33.3%) had low potential for relevant interactions in humans. The percentage of inhibitor drugs turned to negative prediction for OATP inhibition-mediated interactions after performing a clinical study was similar to the ones with p-gp or BCRP inhibitor drugs [43% (13/30 drugs) and 45% (5/11 drugs) for p-gp and BCRP, respectively].

The labels of three, five, and four drugs presented drug interaction potential mediated by OAT1/3, OCT2, and MATE1/2-K inhibition, respectively. While clinical interaction studies confirmed no significant interactions mediated by OAT1 or OAT3 inhibition by two drugs (cefiderocol and letermovir), another two drugs labeled with drug interaction potential mediated by OAT1/3 inhibition [pretomanid and fexinidazole] were not investigated for their interaction potential in humans. Further drug interaction studies for OCT2 inhibition were not available for fexinidazole, erdafitinib, and tafenoquine, leading to a drug interaction statement on the label, with or without PMR. Significant OCT2 inhibition-mediated drug interactions were confirmed for bictegravir and trilaciclib in clinical drug interaction studies, resulting in a statement on the label. No significant changes in blood glucose concentrations were observed in the patients consuming metformin in Phase I and Phase II clinical trials of pemigatinib, and thus pemigatinib was not labeled with OCT2 inhibition-mediated drug interactions, although the R value of pemigatinib on OCT2 inhibition was calculated to be higher than 0.1 (Table 3; classified as an indirect clinical study in no label category). Trilaciclib showed strong inhibitory potency against OCT2, MATE1, and MATE2-K, with IC_50_ values of 0.152, 0.175, and 0.071 µM for each transporter, respectively. The co-administration of trilacicilib with metformin increased the area under the time-plasma concentration curve (AUC) by 65% and the maximum concentration (C_max_) of metformin by 81%. Furthermore, it decreased the renal clearance of metformin by 37%, resulting in a statement on the potential drug interactions with OCT2, MATE1, and MATE2-K inhibition on the label.

The association between the R values of the drugs and their follow-up actions is presented in Figure 2. Drugs with higher R values are likely to create greater alterations in systemic exposure during clinical drug interaction studies, leading to the labeling of drug interactions. In contrast, some drugs with relatively lower R values were labeled for drug interactions when other further actions, such as clinical drug interaction studies, were not performed to clarify the interactions.

### 3.3. In Vitro Substrates and Their Follow-Up Actions

Information on the transporter substrate study was provided with 148, 133, 92, 94, 61, 61, 60, 41, and 39 drugs for P-gp, BCRP, OATP1B1, OATP1B3, OAT1, OAT3, OCT2, MATE1, and MATE2-K, respectively (Table 4, Supplemental Table S4). Information on the P-gp and BCRP was accessible for 95.5 and 85.8% of the drugs, respectively, indicating an intensive focus on these two efflux transporters. The hepatic transporters OATP1B1 and OAPT1B3 were studied with more than 50% of the drugs, while less than 40% presented with results of a substrate study on renal transporters, including OAT1, OAT3, OCT2, MATE1, and MATE2-K.

P-gp and BCRP were involved in the transport of 67.6 and 42.9% of drugs across the membrane in vitro, respectively, proving the broad substrate specificity of the transporters. Among these drugs, only 15 and 14% of the substrate drugs were labeled with potential drug interactions mediated by P-gp and BCRP, respectively (Table 5). A clinical interaction study elucidated the low interaction potential of P-gp and BCRP for 19 and five drugs, respectively, and PBPK modeling results were also suggested to diminish the concern regarding drug interactions mediated by P-gp and BCRP for one (upadacitinib) and two (mobocertinib and upadacitinib) drugs, respectively. Highly permeable drugs included in the Biopharmaceutics Classification System (BCS) class I (high permeability/high solubility; 4 p-gp inhibitor drugs and 2 BCRP inhibitor drugs) or II (high permeability/low solubility; 8 p-gp inhibitor drugs and 3 BCRP inhibitor drugs) tend to receive a waiver for P-gp- or BCRP-mediated drug interactions. The weak involvement of transporters, proven by low efflux or uptake ratio in vitro, was the other frequent reason for not stating concerns about drug interactions on labels. Interestingly, even though lemborexant was implicated as a potentially poor substrate for P-gp, the tested concentration (3 μM) was too high in comparison to the clinically relevant concentration (unbound C_max_ = 10 nM), which may have led to an underestimation of the transporter-mediated efflux of drugs across the membrane; thus, the PMR for the in vitro substrate study at clinically relevant concentrations was issued. This highlights the importance of appropriate test concentrations in transporter substrate studies. 

Less than 20% of the substrate drugs were identified as substrates for hepatic or renal transporters (Table 4). Six drugs were labeled with potential drug interactions with OATP inhibitors, and five of them had significant drug interactions in the clinical interaction study (Table 5). The label for only one substrate drug (baricitinib) states that co-administration with OAT3 inhibitors is not recommended because the AUC of baricitinib increased 2-fold upon co-administration with probenecid. No substrate drug was labeled for potential drug interactions with OCT2, MATE1, or MATE2-K inhibitors.

## 4. Discussion

In this study, we performed a systematic and comprehensive analysis of in vitro transporter substrates and inhibitor drugs and their follow-up actions for in vitro findings. For drugs with labeling on drug interactions, any supporting information on the labeling, such as clinical studies or PBPK modeling, was collected. For drugs with no labeling, even though the R values were higher than the cut-off values, scientific rationales or additional data to claim no necessity of labeling were collected to analyze how the concerns observed in vitro could be resolved. By analyzing various claims and information on drug interactions mediated by transporters, we attempted to gain insights into drug interactions and their labeling. 

Information on in vitro transporter inhibition or substrate was the most available for P-gp (92.9% and 95.5% for inhibition and substrate, respectively). Inhibitory mechanisms on P-gp are diverse, such as competitive inhibition on substrate binding sites or inhibition of ATP hydrolysis, which is a driving force to transport drugs across the membrane [26,27,28], and, thus, various compounds can inhibit P-gp function. In line with this, our study showed that more than one-third of the drugs with available transporter inhibition information showed higher R values than the pre-defined cut-off (i.e., R ≥ 10 for oral drugs and R ≥ 0.1 for parenteral drugs or metabolites), and labels of 40.7% of the drugs suggested caution upon co-administration with P-gp substrates. In addition, more than half of the drugs were in vitro substrates of P-gp, and 15% of these drugs were labeled with caution when co-administered with P-gp inhibitors. These results indicate the significance of P-gp in drug interactions during drug development. 

Less information was available for hepatic and renal transporter substrates compared to that for transporter inhibition, potentially because drug developers do not necessitate the determination of whether the drugs are substrates for all transporters; however, most drugs should be evaluated for their inhibitory effects on transporters. For example, FDA guidelines recommend determining whether drugs are substrates of OATP1B1 and OATP1B3 when the drug’s hepatic uptake or elimination is significant or when the drug’s uptake into the liver is clinically important. In the case of renal transporters, it is suggested that a substrate study should be performed when active renal secretion is significant for drugs. The metabolism of drugs in the liver is a relatively common pathway in drug elimination; thus, more drugs were tested as substrates of hepatic transporters than renal transporters (59.4–60.6% of drugs available for information on OATP1B1/OATP1B3 substrate study versus 25.2–39.4% of drugs available for information on OAT1/OAT3/OCT2/MATE1/MATE2-K substrate study, respectively). 

Clinical drug interaction studies are frequent follow-ups for in vitro transporter inhibitors or substrates to investigate their clinical implications. The changes in systemic exposure upon co-administration provided direct information for the determination of labeling. However, various factors, such as drug solubility or permeability, are considered before proceeding with clinical drug interaction studies. For example, the R value of pemigatinib was above the cut-off for P-gp inhibition (i.e., 10), but the potential drug interaction was not mentioned in the label. It was claimed that pemigatinib has low solubility with high permeability (BCS Class II) and is poorly soluble at pH above 2. Based on this assumption, the gut concentration/IC_50_ ratio is likely <10 for the majority of the gastrointestinal tract, and the inhibitory effect of pemigatinib on P-gp is unlikely to cause a clinically meaningful change in exposure to P-gp substrates. The similar claims were also suggested for BCRP inhibitors (tivozanib and doravirine), and concerns regarding drug interactions with BCRP inhibitors were not described in the labels. Permeability is a frequently suggested property of P-gp and BCRP substrate drugs to explain the low potential of drug interactions. The contribution of active transport to the process of intestinal absorption is likely to be low when the drug is highly permeable. Fourteen and seven drugs were claimed to have a low potential for P-gp- and BCRP-mediated drug interactions due to their high permeability and, hence, were not labeled for the interactions. In addition, several drugs categorized as “not mentioned,” of which information is not available for the absence of a label, are highly permeable drugs; some of these drugs might be exempted from clinical interaction study for the same reason. Of course, the acceptance of this claim may also depend on other factors, such as safety concerns regarding other tissues expressing P-gp and BCRP (e.g., kidney and brain). 

The drug regimen is one of the factors considered to determine whether to describe the drug interaction potential on a label. Remdesivir, approved for the treatment of COVID-19 in 2020, showed R values higher than 0.1 for OATP1B1 and OATP1B3 inhibition, whereas it was not labeled for OATP inhibition, considering its short treatment duration (up to 10 days) and short predicted effect on substrate drugs (up to 4 h only on day one). Similarly, the FDA accepted the claim that a short dosing duration of amisulpride (one day), remdesivir (up to 10 days), and lefamulin (five to seven days) will minimize the potential for drug interactions despite R values higher than the cut-off for MATE1 or MATE2-K inhibition. These drugs are finally not labeled with interactions mediated by MATE1 and MATE2-K inhibition. 

We collected and reviewed information on the interactions of drugs approved for the last five years (2017–2021). The FDA released updated draft guidance on in vitro drug interaction studies in 2017, which contains the study on MATE1 and MATE2-K. Application of this guidance to the drugs approved in 2017 and early 2018 may be untimely; thus, the information available on MATE transporters is less for the drugs approved during this period. Thereafter, information on MATE transporters in NDA documents increased, and the percentage of drugs with available information on MATE transporters was comparable to that of other renal transporters (i.e., OAT1, OAT3, and OCT2). Nevertheless, as the contribution of MATE transporters to drug pharmacokinetics is not well understood compared to the other transporters, information on MATE-specific drug interactions is still rare.

In this study, we attempted to analyze the association between R values of drugs and their follow-up actions, including labeling. We observed that some drugs with relatively lower R values were labeled for drug interactions when other further actions, such as a clinical drug interaction study, was not performed to clarify these interactions. The possibility of no labeling of drug interactions may exist when performing a clinical drug interaction study, which may allow drug use in a wider range of patients. Further analysis using more data available from other regulatory agencies (i.e., the European Medicines Agency and Japan’s Pharmaceuticals and Medical Devices Agency) would be helpful to determine the definite association between R values and follow-up actions. 

We collected information on drug interactions from the NDA review documents and drug labels accessible on the FDA website. FDA review documents reflect the current position of the FDA the best and also contain claims suggested by sponsors. When information on whether the FDA acceptance of sponsor claims is available, we reflected the FDA’s position in our analysis. However, only the sponsor’s claim was reflected in the analysis when the FDA’s conclusion was not presented in the document. Therefore, some information may not reflect the FDA’s opinion, which finally leads to a statement on labels, limiting the accuracy of the analysis in this study. 

Information on drug interactions is invaluable for safe and effective disease treatment. However, unnecessary information or excessive caution regarding drug interactions may limit drug use and thereby reduce the benefit of drugs. The evaluation of potential drug interactions, considering various drug properties and clinical practice, is important. A step-by step approach of various actions sequentially may be useful for more efficient drug development. This study that analyzed follow-up actions on in vitro transporter inhibitors and substrate drugs will assist drug developers in approaching drug interactions more comprehensively and strategically.

## 5. Conclusions

There are several follow-up actions for the in vitro findings on transporter-mediated drug interactions. Clinical drug interaction studies are a frequent way to help determine drug interaction potential. PBPK modeling or the suggestion of scientific rationales based on physicochemical properties or drug regimens also supports the determination of statements on drug interactions on labels. Understanding the type and details of PMR issued by the FDA would also be helpful for efficient drug development. The systematic and comprehensive analysis in this study will provide insight into the management of in vitro findings for transporter substrates or inhibitors. 

## Figures and Tables

**Table 1:** The number of drugs approved by FDA’s CDER between 2017 and 2021 and drugs included for analysis in this study.

Year	No. of drugs approved by FDA’s CDER	No. of drugs included in further analysis
2017	46	28
2018	59	40
2019	48	28
2020	53	30
2021	50	29
Total	256	155

**Table 2:** Drugs with information available on transporter inhibitors and drugs that meet the pre-defined cut-off for potential clinical drug interactions.

The number of drugs with available information (%) ^a^									
Year	P-gp	BCRP	OATP1B1	OATP1B3	OAT1	OAT3	OCT2	MATE1	MATE2-K
2017	28 (100%)	25 (89.3%)	24 (85.7%)	24 (85.7%)	24 (85.7%)	22 (78.6%)	22 (78.6%)	7 (25%)	7 (25%)
2018	35 (87.5%)	34 (85%)	33 (82.5%)	32 (80%)	34 (85%)	34 (85%)	35 (87.5%)	24 (60%)	22 (55%)
2019	27 (96.4%)	26 (92.9%)	28 (100%)	28 (100%)	28 (100%)	28 (100%)	28 (100%)	24 (85.7%)	24 (85.7%)
2020	27 (90%)	26 (86.7%)	27 (90%)	26 (86.7%)	25 (83.3%)	25 (83.3%)	27 (90%)	28 (93.3%)	26 (86.7%)
2021	27 (93.1%)	27 (93.1%)	25 (86.2%)	25 (86.2%)	24 (82.8%)	24 (82.8%)	25 (86.2%)	25 (86.2%)	25 (86.2%)
Total	144 (92.9%)	138 (89%)	137 (88.4%)	135 (87.1%)	135 (87.1%)	133 (85.8%)	137 (88.4%)	108 (69.7%)	104 (67.1%)
**The number of inhibitor drugs with R value above the cut-off (%) ^b^**									
**Year**	**P-gp**	**BCRP**	**OATP1B1**	**OATP1B3**	**OAT1**	**OAT3**	**OCT2**	**MATE1**	**MATE2-K**
2017	10 (35.7%)	13 (52%)	5 (20.8%)	4 (16.7%)	0 (0%)	2 (9.1%)	2 (9.1%)	3 (42.9%)	2 (28.6%)
2018	15 (42.9%)	12 (35.3%)	5 (15.2%)	4 (12.5%)	1 (2.9%)	4 (11.8%)	4 (11.4%)	2 (8.3%)	2 (9.1%)
2019	11 (40.7%)	8 (30.8%)	2 (7.1%)	2 (7.1%)	2 (7.1%)	4 (14.3%)	2 (7.1%)	2 (8.3%)	2 (8.3%)
2020	9 (33.3%)	10 (38.5%)	3 (11.1%)	3 (11.5%)	0 (0%)	1 (4%)	2 (7.4%)	8 (28.6%)	5 (19.2%)
2021	9 (33.3%)	11 (40.7%)	3 (12%)	4 (16%)	2 (8.3%)	3 (12.5%)	2 (8%)	4 (16%)	5 (20%)
Total	54 (37.5%)	54 (39.1%)	18 (13.1%)	17 (12.6%)	5 (3.7%)	14 (10.5%)	12 (8.8%)	19 (17.6%)	16 (15.4%)

^a^ Percentage values (%) were calculated by dividing the number of drugs with available information by the number of drugs included in the analysis (Table 1). ^b^ Percentage values (%) were calculated by dividing the number of drugs with R values above the pre-defined cut-off values by the number of drugs with available information.

**Table 3:** Follow-up actions for in vitro transporter inhibitors with R values above the pre-defined cut-off values.

Category	The number of drugs (%)					
	P-gp	BCRP	OATP1B1/1B3	OAT1/3	OCT2	MATE1/2-K
Label	22 (40.7%)	13 (24.1%)	8 (40%)	3 (21.4%)	5 (41.7%)	4 (16%)
Label (no other study/no PMR)	3 (5.6%)	4 (7.4%)	1 (5%)	2 (14.3%)	2 (16.7%)	2 (8%)
Label/clinical PK	17 (31.5%)	6 (11.1%)	6 (30%)	0 (0%)	2 (16.7%)	1 (4%)
Label/PBPK	0 (0%)	0 (0%)	0 (0%)	1 (7.1)	0 (0%)	0 (0%)
Label/PMR (clinical PK)	2 (3.7%)	3 (5.6%)	1 (5%)	0 (0%)	1 (8.3%)	0 (0%)
Label/indirect clinical study	0 (0%)	0 (0%)	0 (0%)	0 (0%)	0 (0%)	1 (4%)
No label	32 (59.3%)	41 (75.9%)	12 (60%)	11 (78.6%)	7 (58.3%)	21 (84%)
Clinical PK	13 (24.1%)	5 (9.3%)	3 (15%)	2 (14.3%)	2 (16.7%)	5 (20%)
PBPK	2 (3.7%)	0 (0%)	1 (5%)	1 (7.1%)	1 (8.3%)	0 (0%)
PMR	5 (9.3%)	7 (13%)	3 (15%)	0 (0%)	1 (8.3%)	3 (12%)
PMR (clinical PK)	5 (9.3%)	6 (11.1%)	2 (10%)	0 (0%)	1 (8.3%)	3 (12%)
PMR (PBPK)	0 (0%)	1 (1.9%)	1 (5%)	0 (0%)	0 (0%)	0 (0%)
Etc	2 (3.7%)	7 (13%)	1 (5%)	1 (7.1%)	1 (8.3%)	5 (20%)
Indirect clinical study	0 (0%)	4 (7.4%)	0 (0%)	0 (0%)	1 (8.3%)	1 (4%)
Short dosing duration	0 (0%)	0 (0%)	1 (5%)	0 (0%)	0 (0%)	3 (12%)
Low solubility	1 (1.9%)	2 (3.7%)	0 (0%)	0 (0%)	0 (0%)	0 (0%)
No contact	1 (1.9%)	1 (1.9%)	0 (0%)	0 (0%)	0 (0%)	0 (0%)
Static mechanistic model	0 (0%)	0 (0%)	0 (0%)	1 (7.1%)	0 (0%)	0 (0%)
No concomitant medication	0 (0%)	0 (0%)	0 (0%)	0 (0%)	0 (0%)	1 (4%)
Not mentioned	10 (18.5%)	22 (40.7%)	4 (20%)	7 (50%)	2 (16.7%)	8 (32%)
Total	54 (100%)	54 (100%)	20 (100%)	14 (100%)	12 (100%)	25 (100%)

PBPK, physiologically based pharmacokinetics; PK, pharmacokinetics; PMR, post-marketing requirement.

**Table 4:** Drugs with available information on transporter substrates and in vitro transporter substrate drugs.

The number of drugs with available information (%) ^a^									
Year	P-gp	BCRP	OATP1B1	OATP1B3	OAT1	OAT3	OCT2	MATE1	MATE2-K
2017	28 (100%)	25 (89.3%)	17 (60.7%)	18 (64.3%)	16 (57.1%)	14 (50%)	14 (50%)	3 (10.7%)	2 (7.1%)
2018	35 (87.5%)	31 (77.5%)	25 (62.5%)	25 (62.5%)	13 (32.5%)	14 (35%)	14 (35%)	7 (17.5%)	7 (17.5%)
2019	28 (100%)	26 (92.9%)	9 (32.1%)	9 (32.1%)	12 (42.9%)	13 (46.4%)	11 (39.3%)	10 (35.7%)	11 (39.3%)
2020	29 (96.7%)	25 (83.3%)	18 (60%)	19 (63.3%)	9 (30%)	9 (30%)	11 (36.7%)	12 (40%)	10 (33.3%)
2021	28 (96.6%)	26 (89.7%)	23 (79.3%)	23 (79.3%)	11 (37.9%)	11 (37.9%)	10 (34.5%)	9 (31%)	9 (31%)
Total	148 (95.5%)	133 (85.8%)	92 (59.4%)	94 (60.6%)	61 (39.4%)	61 (39.4%)	60 (38.7%)	41 (26.5%)	39 (25.2%)
**The number of in vitro transporter substrate drugs (%) ^b^**									
**Year**	**P-gp**	**BCRP**	**OATP1B1**	**OATP1B3**	**OAT1**	**OAT3**	**OCT2**	**MATE1**	**MATE2-K**
2017	18 (64.3%)	11 (44%)	3 (17.6%)	3 (16.7%)	2 (12.5%)	3 (21.4%)	0 (0%)	0 (0%)	0 (0%)
2018	25 (71.4%)	14 (45.2%)	4 (16%)	3 (12%)	0 (0%)	1 (7.1%)	0 (0%)	0 (0%)	1 (14.3%)
2019	14 (50%)	7 (26.9%)	3 (33.3%)	2 (22.2%)	1 (8.3%)	1 (7.7%)	1 (9.1%)	2 (20%)	2 (18.2%)
2020	25 (86.2%)	16 (64%)	1 (5.6%)	2 (10.5%)	0 (0%)	1 (11.1%)	0 (0%)	1 (8.3%)	1 (10%)
2021	18 (64.3%)	9 (34.6%)	4 (17.4%)	4 (17.4%)	2 (18.2%)	2 (18.2%)	0 (0%)	1 (11.1%)	1 (11.1%)
Total	100 (67.6%)	57 (42.9%)	15 (16.3%)	14 (14.9%)	5 (8.2%)	8 (13.1%)	1 (1.7%)	4 (9.8%)	5 (12.8%)

^a^ Percentage values (%) were calculated by dividing the number of drugs with available information by the number of drugs included in the analysis (Table 1). ^b^ Percentage values (%) were calculated by dividing the number of drugs with R values above the pre-defined cut-off values by the number of drugs.

**Table 5:** Follow-up actions for in vitro transporter substrates.

Category	The number of drugs (%)					
	P-gp	BCRP	OATP1B1/1B3	OAT1/3	OCT2	MATE1 /2-K
Label	15 (15%)	8 (14%)	6 (40%)	1 (10%)	0 (0%)	0 (0%)
Label	4 (4%)	1 (6.7%)	1 (6.7%)	0 (0%)	0 (0%)	0 (0%)
Label/clinical PK	7 (7%)	5 (33.3%)	5 (33.3%)	1 (10%)	0 (0%)	0 (0%)
Label/PMR (clinical PK)	4 (4%)	1 (1.8%)	0 (0%)	0 (0%)	0 (0%)	0 (0%)
No label	85 (85%)	49 (86%)	9 (60%)	9 (90%)	1 (100%)	7 (100%)
Clinical PK	19 (19%)	5 (8.8%)	1 (6.7%)	1 (10%)	0 (0%)	0 (0%)
PBPK	1 (1%)	2 (3.5%)	0 (0%)	0 (0%)	0 (0%)	0 (0%)
PMR	3 (3%)	1 (1.8%)	1 (6.7%)	0 (0%)	0 (0%)	0 (0%)
PMR (clinical PK)	2 (2%)	1 (1.8%)	1 (6.7%)	0 (0%)	0 (0%)	0 (0%)
PMR (in vitro study)	1 (1%)	0 (0%)	0 (0%)	0 (0%)	0 (0%)	0 (0%)
Etc	38 (38%)	22 (38.6%)	4 (26.7%)	8 (80%)	1 (100%)	5 (71.4%)
High permeability	14 (14%)	7 (12.3%)	0 (0%)	0 (0%)	0 (0%)	0 (0%)
Weak substrate	7 (7%)	3 (5.3%)	2 (13.3%)	2 (20%)	1 (100%)	2 (28.6%)
Not major elimination route	1 (1%)	1 (1.8%)	2 (13.3%)	2 (20%)	0 (0%)	1 (14.3%)
Saturation	2 (2%)	1 (1.8%)	0 (0%)	0 (0%)	0 (0%)	0 (0%)
Wide safety range	1 (1%)	2 (3.5%)	0 (0%)	0 (0%)	0 (0%)	0 (0%)
Indirect clinical study	2 (2%)	0 (0%)	0 (0%)	0 (0%)	0 (0%)	1 (14.3%)
IV dosing/no safety concern	1 (1%)	1 (1.8%)	0 (0%)	0 (0%)	0 (0%)	0 (0%)
Low solubility	1 (1%)	0 (0%)	0 (0%)	0 (0%)	0 (0%)	0 (0%)
Short dosing duration	0 (0%)	0 (0%)	0 (0%)	0 (0%)	0 (0%)	1 (14.3%)
Not mentioned	33 (33%)	26 (45.6%)	3 (20%)	4 (40%)	0 (0%)	2 (28.6%)
Total	100 (100%)	57 (100%)	15 (100%)	10 (100%)	1 (100%)	7 (100%)

IV, intravenous; PBPK, physiologically based pharmacokinetics; PK, pharmacokinetics; PMR, post-marketing requirement.

## Data Availability

Not applicable.

## Supplementary Materials

The following supporting information can be downloaded at: https://www.mdpi.com/article/10.3390/pharmaceutics14102078/s1, Table S1. List of drugs involved in the analysis of this study; Table S2. R values calculated by basic method; Table S3. Follow-up actions for in vitro transporter inhibitor drugs or rationales for no labeling; Table S4. Follow-up actions for in vitro transporter substrate drugs or rationales for no labeling.
