# Supplementary material for: Findings on In Vitro Transporter-Mediated Drug Interactions and Their Follow-Up Actions for Labeling: Analysis of Drugs Approved by US FDA between 2017 and 2021
Source: Pharmaceutics. 2022 Sep 29;14(10):2078. doi: 10.3390/pharmaceutics14102078 (PMC9607947; doi:10.3390/pharmaceutics14102078)
Supplement: Supplementary file 1 [file pharmaceutics-14-02078-s001.zip › pharmaceutics-1894852-supplementary.pdf]

Supplemental Table S1. List of drugs involved in the analysis of this study

Drug name	Active ingredient	Approval date	FDA-approved use on approval date
Trulance	plecanatide	1/19/2017	To treat Chronic Idiopathic Constipation (CIC) in adult patients.
Parsabiv	etelcalcetide	2/7/2017	To treat secondary hyperparathyroidism in adult patients with chronic kidney disease undergoing dialysis
Emflaza	deflazacort	2/9/2017	To treat patients age 5 years and older with Duchenne muscular dystrophy (DMD)
Xermelo	telotristat ethyl	2/28/2017	To treat carcinoid syndrome diarrhea
Kisqali	ribociclib	3/13/2017	To treat postmenopausal women with a type of advanced breast cancer
Xadago	safinamide	3/21/2017	To treat Parkinson’s disease
Symproic	naldemedine	3/23/2017	For the treatment of opioid-induced constipation
Zejula	niraparib	3/27/2017	For the maintenance treatment for recurrent epithelial ovarian, fallopian tube or primary peritoneal cancers
Austedo	deutetrabenazine	4/3/2017	For the treatment of chorea associated with Huntington’s disease
Ingrezza	valbenazine	4/11/2017	To treat adults with tardive dyskinesia
Alunbrig	brigatinib	4/28/2017	To treat patients with anaplastic lymphoma kinase (ALK)-positive metastatic non-small cell lung cancer (NSCLC) who have progressed on or are intolerant to crizotinib
Rydapt	midostaurin	4/28/2017	To treat acute myeloid leukemia, advanced systemic mastocytosis
Radicava	edaravone	5/5/2017	To treat patients with amyotrophic lateral sclerosis (ALS)
Baxdela	delafloxacin	6/19/2017	To treat patients with acute bacterial skin infections
Bevyxxa	betrixaban	6/23/2017	For the prophylaxis of venous thromboembolism (VTE) in adult patients hospitalized for an acute medical illness
Nerlynx	neratinib maleate	7/17/2017	To reduce the risk of breast cancer returning
Vosevi	sofosbuvir, velpatasvir and voxilaprevir	7/18/2017	To treat adults with chronic hepatitis C virus
Idhifa	enasidenib	8/1/2017	To treat relapsed or refractory acute myeloid leukemia
Mavyret	glecaprevir and pibrentasvir	8/3/2017	To treat adults with chronic hepatitis C virus
Vabomere	meropenem and vaborbactam	8/29/2017	To treat adults with complicated urinary tract infections
Benznidazole	benznidazole	8/29/2017	To treat children ages 2 to 12 years old with Chagas disease
Aliqopa	copanlisib	9/14/2017	To treat adults with relapsed follicular lymphoma
Solosec	secnidazole	9/15/2017	To treat bacterial vaginosis
Verzenio	abemaciclib	9/28/2017	To treat certain advanced or metastatic breast cancers
Calquence	acalabrutinib	10/31/2017	To treat adults with mantle cell lymphoma
Prevymis	letermovir	11/8/2017	To prevent infection after bone marrow transplant
Steglatro	ertugliflozin	12/19/2017	To improve glycemic control in adults with type 2 diabetes mellitus
Macrilen	macimorelin acetate	12/20/2017	For the diagnosis of adult growth hormone deficiency
Lutathera	lutetium Lu 177 dotatate	1/26/2018	To treat a type of cancer that affects the pancreas or gastrointestinal tract called gastroenteropancreatic neuroendocrine tumors (GEP-NETs).
Biktarvy	bictegravir, embitcitabine, and tenofovir alafenamide	2/7/2018	To treat infection in adults who have no antiretroviral treatment history or to replace the current antiretroviral regimen
Symdeko	tezacaftor andivacaftor	2/12/2018	To treat cystic fibrosis in patients age 12 years and older

Drug name	Active ingredient	Approval date	FDA-approved use on approval date
Erleada	apalutamide	2/14/2018	To treat a certain type of prostate cancer using novel clinical trial endpoint
Tavalisse	fostamatinib	4/17/2018	To treat thrombocytopenia in adult patients with persistent or chronic immune thrombocytopenia (ITP)
Akynzeo	fosnetupitant and palonosetron	4/19/2018	To prevent acute and delayed nausea and vomiting associated with initial and repeat courses of highly emetogenic cancer chemotherapy
Lucemyra	lofexidine hydrochloride	5/16/2018	For the non-opioid treatment for management of opioid withdrawal symptoms in adults
Lokelma	sodium zirconium cyclosilicate	5/18/2018	To treat hyperkalemia
Doptelet	avatrombopag	5/21/2018	To treat low blood platelet count (thrombocytopenia) in adults with chronic liver disease who are scheduled to undergo a medical or dental procedure
Olumiant	baricitinib	5/31/2018	To treat moderately to severely active rheumatoid arthritis
Moxidectin	moxidectin	6/13/2018	To treat onchocerciasis due to Onchocerca volvulus in patients aged 12 years and older
Epidioloex	cannabidiol	6/25/2018	To treat rare, severe forms of epilepsy
Zemdri	plazomicin	6/25/2018	To treat adults with complicated urinary tract infections
Mektovi	binimetinib	6/27/2018	To treat unresectable or metastatic melanoma
Braftovi	encorafenib	6/27/2018	To treat unresectable or metastatic melanoma
Tpoxx	tecovirimat	7/13/2018	To treat smallpox
Tibsovo	ivosidenib	7/20/2018	To treat patients with relapsed or refractory acute myeloid leukemia
Krintafel	tafenoquine	7/20/2018	For the radical cure (prevention of relapse) of Plasmodium vivax malaria
Orilissa	elagolix sodium	7/23/2018	For the management of moderate to severe pain associated with endometriosis
Omegaven	fish oil triglycerides	7/27/2018	As a source of calories and fatty acids in pediatric patients with parenteral nutrition-associated cholestasis
Mulpleta	lusutrombopag	7/31/2018	To treat thrombocytopenia in adult patients with chronic liver disease who are scheduled to undergo a procedure
Annovera	segesterone acetate and ethinyl estradiol vaginal system	8/10/2018	New vaginal ring used to prevent pregnancy for an entire year
Galafold	migalastat	8/10/2018	To treat adults with Fabry disease.
Diacomit	stiripentol	8/20/2018	To treat seizures associated with Dravet syndrome in patients 2 years of age and older taking clobazam
Xerava	eravacycline	8/27/2018	To treat complicated intra-abdominal infections in patients 18 years of age and older
Pifeltro	doravirine	8/30/2018	To treat HIV-1 infection in adult patients
Copiktra	duvelisib	9/24/2018	To treat relapsed or refractory chronic lymphocytic leukemia, small lymphocytic lymphoma and follicular lymphoma
Vizimpro	dacomitinib	9/27/2018	To treat metastatic non-small-cell lung cancer
Seysara	sarecycline	10/1/2018	To treat inflammatory lesions of non-nodular moderate to severe acne vulgaris in patients 9 years of age and older
Nuzyra	omadacycline	10/2/2018	To treat community-acquired bacterial pneumonia and acute bacterial skin and skin structure infections

Drug name	Active ingredient	Approval date	FDA-approved use on approval date
Talzenna	talazoparib	10/16/2018	To treat locally advanced or metastatic breast cancer patients with a germline BRCA mutation.
Xofluza	baloxavir marboxil	10/24/2018	To treat acute uncomplicated influenza in patients who have been symptomatic for no more than 48 hours.
Lorbrena	lorlatinib	11/2/2018	To treat patients with anaplastic lymphoma kinase (ALK)-positive metastatic non-small cell lung cancer
Yupelri	revefenacin	11/9/2018	To treat patients with chronic obstructive pulmonary disease (COPD)
Aemcolo	rifamycin	11/16/2018	To treat travelers' diarrhea
Daurismo	glasdegib	11/21/2018	To treat newly-diagnosed acute myeloid leukemia (AML) in adult patients
Vitrakvi	larotrectinib	11/26/2018	To treat patients whose cancers have a specific genetic feature (biomarker)
Firdapse	amifampridine	11/28/2018	To treat Lambert-Eaton myasthenic syndrome (LEMS) in adults
Xospata	gilteritinib	11/28/2018	To treat patients who have relapsed or refractory acute myeloid leukemia (AML)
Motegrity	prucalopride	12/14/2018	To treat chronic idiopathic constipation
Egaten	triclabendazole	2/13/2019	To treat fascioliasis, a parasitic infestation caused by two species of flatworms or trematodes that mainly the affect the liver, sometimes referred to as "liver flukes"
Zulresso	brexanolone	3/19/2019	To treat postpartum depression (PPD) in adult women
Sunosi	solriamfetol	3/20/2019	To treat excessive sleepiness in adult patients with narcolepsy or obstructive sleep apnea
Mayzent	siponimod	3/26/2019	To treat adults with relapsing forms of multiple sclerosis
Balversa	erdafitinib	4/12/2019	To treat adult patients with locally advanced or metastatic bladder cancer
Vyndaqel	tafamidis meglumine	5/3/2019	To treat heart disease (cardiomyopathy) caused by transthyretin mediated amyloidosis (ATTR-CM) in adults
Piqray	alpelisib	5/24/2019	To treat breast cancer
Xpovio	selinexor	7/3/2019	To treat adult patients with relapsed or refractory multiple myeloma (RRMM)
Recarbrio	imipenem, cilastatin and relebactam	7/16/2019	To treat complicated urinary tract and complicated intra-abdominal infections
Nubeqa	darolutamide	7/30/2019	To treat adult patients with non-metastatic castration resistant prostate cancer
Turalio	pexidartinib	8/2/2019	To treat adult patients with symptomatic tenosynovial giant cell tumor
Pretomanid	pretomanid	8/14/2019	For treatment-resistant forms of tuberculosis that affects the lungs
Wakix	pitolisant	8/14/2019	To treat excessive daytime sleepiness (EDS) in adult patients with narcolepsy
Rozlytrek	entrectinib	8/15/2019	To treat adult patients with metastatic non-small cell lung cancer (NSCLC) whose tumors are ROS1-positive
Inrebic	fedratinib	8/16/2019	To treat adult patients with intermediate-2 or high-risk primary or secondary myelofibrosis
Rinvoq	upadacitinib	8/16/2019	To treat adults with moderately to severely active rheumatoid arthritis
Xenleta	lefamulin	8/19/2019	To treat adults with community-acquired bacterial pneumonia
Nourianz	istradefylline	8/27/2019	To treat adult patients with Parkinson's disease experiencing "off" episodes
Ibsrela	tenapanor	9/12/2019	To treat irritable bowel syndrome with constipation in adults.
Reyvow	lasmiditan	10/11/2019	For the acute treatment of migraine with or without aura, in adults
Trikafta	elexacaftor, ivacaftor, and tezacaftor	10/21/2019	To treat patients 12 years of age and older with the most common gene mutation that causes cystic fibrosis

Drug name	Active ingredient	Approval date	FDA-approved use on approval date
Brukinsa	zanubrutinib	11/14/2019	To treat certain patients with mantle cell lymphoma, a form of blood cancer
Fetroja	cefiderocol	11/14/2019	To treat patients with complicated urinary tract infections who have limited or no alternative treatment options
Xcopri	cenobamate	11/21/2019	To treat partial onset seizures
Oxbryta	voxelotor	11/25/2019	To treat sickle cell disease
Caplyta	lumateperone tosylate	12/20/2019	To treat schizophrenia
Dayvigo	lemborexant	12/20/2019	To treat insomnia
Enhertu	fam-trastuzumab deruxtecan-nxki	12/20/2019	To treat metastatic breast cancer
Ubrelvy	ubrogepant	12/23/2019	to treat acute treatment of migraine with or without aura in adults
Ayvakit	avapritinib	1/9/2020	To treat adults with unresectable or metastatic gastrointestinal stromal tumor (GIST)
Tazverik	tazemetostat	1/23/2020	To treat epithelioid sarcoma
Pizensy	lactitol	2/12/2020	To treat chronic idiopathic constipation (CIC) in adults
Nexletol	bempedoic acid	2/21/2020	To treat adults with heterozygous familial hypercholesterolemia or established atherosclerotic cardiovascular disease who require additional lowering of LDL-C
Barhemsys	amisulpride	2/26/2020	To help prevent nausea and vomiting after surgery
Nurtec ODT	rimegepant	2/27/2020	To treat migraine
Isturisa	osilodrostat	3/6/2020	To treat adults with Cushing's disease who either cannot undergo pituitary gland surgery or have undergone the surgery but still have the disease
Zeposia	ozanimod	3/25/2020	To treat relapsing forms of multiple sclerosis
Koselugo	selumetinib	4/10/2020	To treat neurofibromatosis type 1, a genetic disorder of the nervous system causing tumors to grow on nerves
Tukysa	tucatinib	4/17/2020	To treat advanced unresectable or metastatic HER2-positive breast cancer
Pemazyre	pemigatinib	4/17/2020	To treat certain patients with cholangiocarcinoma, a rare form of cancer that forms in bile ducts
Ongentys	opicapone	4/24/2020	To treat patients with Parkinson's disease experiencing "off" episodes
Tabrecta	capmatinib	5/6/2020	To treat patients with non-small cell lung cancer
Retevmo	selpercatinib	5/8/2020	To treat lung and thyroid cancers
Qinlock	ripretinib	5/15/2020	To treat advanced gastrointestinal-stromal tumors
Artesunate	artesunate	5/26/2020	To treat severe malaria
Zepzelca	lurbinectedin	6/15/2020	To treat metastatic small cell lung cancer
Dojolvi	triheptanoin	6/30/2020	To treat molecularly long-chain fatty acid oxidation disorders
Byfavo	remimazolam	7/2/2020	For sedation
Rukobia	fostemsavir	7/2/2020	To treat HIV
Inqovi	decitabine and cedazuridine	7/7/2020	To treat adult patients with myelodysplastic syndromes
Lampit	nifurtimox	8/6/2020	To treat Chagas disease in certain pediatric patients younger than age 18
Evrysdi	risdiplam	8/7/2020	To treat spinal muscular atrophy
Olinvyk	oliceridine	8/7/2020	To manage acute pain in certain adults
Gavreto	pralsetinib	9/4/2020	To treat non-small lung cancer
Veklury	remdesivir	10/22/2020	To treat COVID-19

Drug name	Active ingredient	Approval date	FDA-approved use on approval date
Zokinvy	lonafarnib	11/20/2020	To treat rare conditions related to premature aging
Orladeyo	berotralstat	12/3/2020	To treat patients with hereditary angioedema
Orgovyx	relugolix	12/18/2020	To treat advanced prostate cancer
Gemtesa	vibegron	12/23/2020	To treat overactive bladder
Verquvo	vericiguat	1/19/2021	To treat chronic heart failure
Cabenuva	cabotegravir and rilpivirine	1/21/2021	To treat HIV
Lupkynis	voclosporin	1/22/2021	To treat lupus nephritis
Tepmetko	tepotinib	2/3/2021	To treat non-small cell lung cancer
Ukoniq	umbralisib	2/5/2021	For the treatment of certain patients with marginal zone lymphoma and follicular lymphoma
Cosela	trilaciclib	2/12/2021	To mitigate chemotherapy-induced myelosuppression in adult patients with small cell lung cancer
Nulibry	fosdenopterin	2/26/2021	To treat patients with the rare genetic disease molybdenum cofactor deficiency Type A
Pepaxto	melphalan flufenamide	2/26/2021	For the treatment of certain patients with relapsed or refractory multiple myeloma
Azstarys	serdexmethylphenidate and dexmethylphenidate	3/2/2021	For the treatment of Attention Deficit Hyperactivity
Fotivda	tivozanib	3/10/2021	To treat patients with renal cell carcinoma
Ponvory	ponesimod	3/18/2021	To treat patients with relapsing forms of multiple sclerosis
Qelbree	viloxazine	4/2/2021	For the treatment of attention deficit hyperactivity disorder
Nextstellis	drospirenone and estetrol tablets	4/15/2021	To prevent pregnancy
Lumakras	sotorasib	5/28/2021	To treat adults with non-small cell lung cancer whose disease meets certain criteria
Truseltiq	infigratinib	5/28/2021	To treat adults with cholangiocarcinoma whose disease meets certain criteria
Lybalvi	olanzapine and samidorphan	5/28/2021	To treat schizophrenia in adults and certain aspects of bipolar I disorder in adults
Brexafemme	ibrexafungerp	6/1/2021	To treat vulvovaginal candidiasis in adult females and pediatric females who have begun menstruating
Kerendia	finerenone	7/9/2021	To reduce the risk of kidney and heart complications in chronic kidney disease associated with type 2 diabetes
fexinidazole	fexinidazole	7/16/2021	To treat human African trypanosomiasis caused by the parasite Trypanosoma brucei gambiense
Rezurock	belumosudil	7/16/2021	To treat chronic graft-versus-host disease after failure of at least two prior lines of systemic therapy
Bylvay	odevixibat	7/20/2021	To treat pruritus
Welireg	belzutifan	8/13/2021	To treat von Hippel-Lindau disease under certain conditions
Korsuva	difelikefalin	8/23/2021	To treat moderate-to-severe pruritus associated with chronic kidney disease in certain populations
Exkivity	mobocertinib	9/15/2021	To treat locally advanced or metastatic non-small cell lung cancer with epidermal growth factor receptor exon 20 insertion mutations
Qulipta	atogepant	9/28/2021	To prevent episodic migraines
Livmarli	maralixibat	9/29/2021	To treat cholestatic pruritus associated with Alagille syndrome

Drug name	Active ingredient	Approval date	FDA-approved use on approval date
Tavneos	avacopan	10/7/2021	To treat severe active anti-neutrophil cytoplasmic autoantibody-associated vasculitis (granulomatosis with polyangiitis and microscopic polyangiitis) in combination with standard therapy, including glucocorticoids
Scemblix	asciminib	10/29/2021	To treat Philadelphia chromosome-positive chronic myeloid leukemia with disease that meets certain criteria
Livtencity	maribavir	11/23/2021	To treat post-transplant cytomegalovirus (CMV) infection/disease that does not respond (with or without genetic mutations that cause resistance) to available antiviral treatment for CMV

Supplemental Table S2. R values calculated by basic method

(dark shading)	≥10 × R value
(medium shading)	≥ 1× and < 10 × R value
(light shading)	≥ 0.1× and < 1× R value
(no shading)	N/A

Drug name	Active Ingredient	R values calculated by basic method										
		P-gp (I_{gut})	P-gp (I_{max})	BCRP (I_{gut})	BCRP (I_{max})	OATP1B1	OATP1B3	OAT1	OAT3	OCT2	MATE1	MAEK2-K
Aemcolo	rifamycin	≥10 × R value		≥ 1× and < 10 × R value		≥ 0.1× and < 1× R value	≥ 0.1× and < 1× R value					
Akynzeo	fosnetupitant and palonosetron		≥ 1× and < 10 × R value			≥ 0.1× and < 1× R value	≥ 0.1× and < 1× R value		≥ 0.1× and < 1× R value	≥ 0.1× and < 1× R value	≥ 0.1× and < 1× R value	≥ 0.1× and < 1× R value
Aliqopa	copanlisib		≥ 0.1× and < 1× R value		≥ 0.1× and < 1× R value						≥ 0.1× and < 1× R value	≥10 × R value
Alunbrig	brigatinib	≥10 × R value		≥10 × R value							≥ 1× and < 10 × R value	≥ 0.1× and < 1× R value
Annovera	segesterone acetate and ethinyl estradiol vaginal system											
Artesunate	artesunate					≥ 0.1× and < 1× R value						
Austedo	deutetrabenazine											
Ayvakit	avapritinib	≥10 × R value		≥10 × R value							≥ 0.1× and < 1× R value	≥ 1× and < 10 × R value
Azstarys	serdexmethylphenidate and dexmethylphenidate											
Balversa	erdafitinib	≥ 1× and < 10 × R value								≥ 1× and < 10 × R value		
Barhemsys	amisulpride										≥ 1× and < 10 × R value	≥ 0.1× and < 1× R value
Baxdela	delafloxacin									≥ 0.1× and < 1× R value		
Benznidazole	benznidazole								≥ 1× and < 10 × R value			
Bevyxxa	betrixaban	≥10 × R value		≥10 × R value								
Biktarvy	bictegravir, embitcitabine, and tenofovir alafenamide									≥ 1× and < 10 × R value	≥ 0.1× and < 1× R value	
Braftovi	encorafenib	≥ 1× and < 10 × R value		≥10 × R value		≥10 × R value	≥10 × R value	≥ 1× and < 10 × R value	≥10 × R value	≥ 1× and < 10 × R value		
Brexafemme	ibrexafungerp	≥10 × R value		≥ 0.1× and < 1× R value		≥ 0.1× and < 1× R value	≥10 × R value					
Brukinsa	zanubrutinib	≥10 × R value		≥10 × R value						≥ 0.1× and < 1× R value		
Byfavo	remimazolam					≥ 0.1× and < 1× R value			≥ 0.1× and < 1× R value			
Bylvay	odevixibat	≥ 0.1× and < 1× R value		≥ 0.1× and < 1× R value		≥ 0.1× and < 1× R value						
Cabenuva	cabotegravir and rilpivirine			≥ 0.1× and < 1× R value				≥ 1× and < 10 × R value	≥ 1× and < 10 × R value		≥ 0.1× and < 1× R value	≥ 0.1× and < 1× R value
Calquence	acalabrutinib	≥ 0.1× and < 1× R value		≥ 1× and < 10 × R value								
Caplyta	lumateperone tosylate					≥ 0.1× and < 1× R value	≥ 0.1× and < 1× R value					

Drug name	Active Ingredient	R values calculated by basic method										
		P-gp (I_{gut})	P-gp (I_{max})	BCRP (I_{gut})	BCRP (I_{max})	OATP1B1	OATP1B3	OAT1	OAT3	OCT2	MATE1	MAEK2-K
Copiktra	duvelisib											
Cosela	trilaciclib											
Daurismo	glasdegib											
Dayvigo	lemborexant											
Diacomit	stiripentol											
Dojolvi	triheptanoin											
Doptelet	avatrombopag											
Egaten	triclabendazole											
Emflaza	deflazacort											
Enhertu	fam-trastuzumab deruxtecan-nxki											
Epidioloex	cannabidiol											
Erleada	apalutamide											
Evrysdi	risdiplam											
Exkivity	mobocertinib											
Fetroja	cefiderocol											
Fexinidazole	fexinidazole											
Firdapse	amifampridine											
Fotivda	tivozanib											
Galafold	migalastat											
Gavreto	pralsetinib											
Gemtesa	vibegron											
Ibsrela	tenapanor											
Idhifa	enasidenib											
Ingrezza	valbenazine											
Inqovi	decitabine and cedazuridine											
Inrebic	fedratinib											
Isturisa	osilodrostat											
Kerendia	finerenone											
Kisqali	ribociclib											
Korsuva	difelikefalin											
Koselugo	selumetinib											
Krintafel	tafenoquine											
Lampit	nifurtimox											
Livmarli	maralixibat											
Livtencity	maribavir											
Lokelma	sodium zirconium cyclosilicate											

Drug name	Active Ingredient	R values calculated by basic method										
		P-gp (I_{gut})	P-gp (I_{max})	BCRP (I_{gut})	BCRP (I_{max})	OATP1B1	OATP1B3	OAT1	OAT3	OCT2	MATE1	MAEK2-K
Lorbrena	lorlatinib											
Lucemyra	lofexidine hydrochloride											
Lumakras	sotorasib											
Lupkynis	voclosporin											
Lutathera	lutetium Lu 177 dotatate											
Lybalvi	olanzapine and samidorphan											
Macrilen	macimorelin acetate											
Mavyret	glecaprevir and pibrentasvir											
Mayzent	siponimod											
Mektovi	binimetinib											
Motegrity	prucalopride											
Moxidectin	moxidectin											
Mulpleta	lusutrombopag											
Nerlynx	neratinib maleate											
Nexletol	bempedoic acid											
Nextstellis	drospirenone and estetrol tablets											
Nourianz	istradefylline											
Nubeqa	darolutamide											
Nulibry	fosdenopterin											
Nurtec ODT	rimegepant											
Nuzyra	omadacycline											
Olinvyk	oliceridine											
Olumiant	baricitinib											
Omegaven	fish oil triglycerides											
Ongentys	opicapone											
Orgovyx	relugolix											
Orilissa	elagolix sodium											
Orladeyo	berotralstat											
Oxbryta	voxelotor											
Parsabiv	etelcalcetide											
Pemazyre	pemigatinib											
Pepaxto	melphalan flufenamide											
Pifeltro	doravirine											
Piqray	alpelisib											
Pizensy	lactitol											
Ponvory	ponesimod											
pretomanid	pretomanid											

Drug name	Active Ingredient	R values calculated by basic method										
		P-gp (I_{gut})	P-gp (I_{max})	BCRP (I_{gut})	BCRP (I_{max})	OATP1B1	OATP1B3	OAT1	OAT3	OCT2	MATE1	MAEK2-K
Prevymis	letermovir											
Qelbree	viloxazine											
Qinlock	ripretinib											
Qulipta	atogepant											
Radicava	edaravone											
Recarbrio	imipenem, cilastatin and relebactam											
Retevmo	selpercatinib											
Reyvow	lasmiditan											
Rezurock	belumosudil											
Rinvoq	upadacitinib											
Rozlytrek	entrectinib											
Rukobia	fostemsavir											
Rydapt	midostaurin											
Scemblix	asciminib											
Seysara	sarecycline											
Solosec	secnidazole											
Steglatro	ertugliflozin											
Sunosi	solriamfetol											
Symdeko	tezacaftor and ivacaftor											
Symproic	naldemedine											
Tabrecta	capmatinib											
Talzenna	talazoparib											
Tavalisse	fostamatinib											
Tavneos	avacopan											
Tazverik	tazemetostat											
Tepmetko	tepotinib											
Tibsovo	ivosidenib											
Tpoxx	tecovirimat											
Trikafta	elexacaftor, ivacaftor, and tezacaftor											
Trulance	plecanatide											
Truseltiq	infigratinib											
Tukysa	tucatinib											
Turalio	pexidartinib											
Ubrelvy	ubrogepant											
Ukoniq	umbralisib											

Drug name	Active Ingredient	R values calculated by basic method										
		P-gp (I_{gut})	P-gp (I_{max})	BCRP (I_{gut})	BCRP (I_{max})	OATP1B1	OATP1B3	OAT1	OAT3	OCT2	MATE1	MAEK2-K
Vabomere	meropenem and vaborbactam											
Veklury	remdesivir											
Verquvo	vericiguat											
Verzenio	abemaciclib											
Vitrakvi	larotrectinib											
Vizimpro	dacomitinib											
Vosevi	sofosbuvir, velpatasvir and voxilaprevir											
Vyndaqel	tafamidis meglumine											
Wakix	pitolisant											
Welireg	belzutifan											
Xadago	safinamide											
Xcopri	cenobamate											
Xenleta	lefamulin											
Xerava	eravacycline											
Xermelo	telotristat ethyl											
Xofluza	baloxavir marboxil											
Xospata	gilteritinib											
Xpovio	selinexor											
Yupelri	revefenacin											
Zejula	niraparib											
Zemdri	plazomicin											
Zeposia	ozanimod											
Zepzelca	lurbinectedin											
Zokinvy	lonafarnib											
Zulresso	brexanolone											

I_{gut}, the concentration of inhibitors in intestinal fluid; I_{max}, C_{max} of inhibitor drug

Supplemental Table S3. Follow-up actions for *in vitro* transporter inhibitor drugs or rationales for no labeling

Drug name	Active ingredient	P-gp	BCRP	OATP1B1/1B3	OAT1/3	OCT2	MATE1/2-K
Aemcolo	rifamycin	No contact	No contact	N/A	N/A	N/A	N/A
Akynzeo	fosnetupitant and palonosetron	PBPK	N/A	N/A	N/A	N/A	N/A
Aliqopa	copanlisib	N/A	N/A	N/A	N/A	N/A	PMR (clinical PK)
Alunbrig	brigatinib	Not mentioned	Not mentioned	N/A	N/A	N/A	Not mentioned
Ayvakit	avapritinib	Not mentioned	Not mentioned	N/A	N/A	N/A	Not mentioned
Balversa	erdafitinib	Label	N/A	N/A	N/A	Label/PMR (clinical PK)	N/A
Barhemsys	amisulpride	N/A	N/A	N/A	N/A	N/A	Short dosing duration
Benznidazole	benznidazole	N/A	N/A	N/A	Not mentioned	N/A	N/A
Bevyxxa	betrixaban	Clinical PK	Indirect clinical study	N/A	N/A	N/A	N/A
Biktarvy	bictegravir, embitcitabine, and tenofovir alafenamide	N/A	N/A	N/A	N/A	Label/clinical PK	N/A
Braftovi	encorafenib	Not mentioned	Not mentioned	Not mentioned	Not mentioned	Not mentioned	N/A
Brexafemme	ibrexafungerp	Clinical PK	N/A	Clinical PK/short dosing duration	N/A	N/A	N/A
Brukinsa	zanubrutinib	Clinical PK	Clinical PK	N/A	N/A	N/A	N/A
Cabenuva	cabotegravir and rilpivirine	N/A	N/A	N/A	Label/PBPK	N/A	N/A
Calquence	acalabrutinib	N/A	Label	N/A	N/A	N/A	N/A
Cosela	trilaciclib	N/A	Not mentioned	N/A	N/A	Label/clinical PK	Label/clinical PK
Daurismo	glasdegib	Not mentioned	Not mentioned	N/A	N/A	N/A	Not mentioned
Diacomit	stiripentol	Label/PMR (clinical PK)	Label/PMR (clinical PK)	N/A	N/A	N/A	N/A
Doptelet	avatrombopag	N/A	Not mentioned	N/A	Not mentioned	N/A	N/A
Erleada	apalutamide	Clinical PK	Clinical PK	N/A	N/A	PBPK	N/A
Evrysdi	risdiplam	N/A	N/A	N/A	N/A	N/A	Label
Exkivity	mobocertinib	PBPK	PMR (clinical PK)	N/A	N/A	N/A	N/A
Fetroja	cefiderocol	N/A	N/A	N/A	Clinical PK	N/A	N/A
Fexinidazole	fexinidazole	N/A	N/A	N/A	Label	Label	Label
Fotivda	tivozanib	N/A	Low solubility	N/A	N/A	N/A	N/A
Gavreto	pralsetinib	PMR (clinical PK)	PMR (clinical PK)	PMR (clinical PK)	N/A	N/A	PMR (clinical PK)
Idhifa	enasidenib	Label/clinical PK	Label/clinical PK	Label/clinical PK	N/A	N/A	N/A
Ingrezza	valbenazine	Label/clinical PK	N/A	N/A	N/A	N/A	N/A
Inrebic	fedratinib	PMR (clinical PK)	PMR (clinical PK)	N/A	N/A	PMR (clinical PK)	PMR (clinical PK)
Isturisa	osilodrostat	N/A	N/A	N/A	Not mentioned	N/A	Not mentioned
Kerendia	finerenone	N/A	Not mentioned	N/A	N/A	N/A	N/A
Kisqali	ribociclib	N/A	Not mentioned	N/A	N/A	Not mentioned	Not mentioned

Drug name	Active ingredient	P-gp	BCRP	OATP1B1/1B3	OAT1/3	OCT2	MATE1/2-K
Krintafel	tafenoquine	N/A	N/A	N/A	N/A	Label	N/A
Livtencity	maribavir	Label/clinical PK	Label	N/A	N/A	N/A	N/A
Lorbrena	lorlatinib	Not mentioned	Not mentioned	Not mentioned	Not mentioned	N/A	Not mentioned
Lumakras	sotorasib	Label/clinical PK	PMR (clinical PK)	N/A	N/A	N/A	Clinical PK
Lupkynis	voclosporin	Label/clinical PK	N/A	Label/PMR (clinical PK)	N/A	N/A	N/A
Mavyret	glecaprevir and pibrentasvir	Label/clinical PK	Label	Label/clinical PK	N/A	N/A	N/A
Nourianz	istradefylline	Label/clinical PK	N/A	N/A	N/A	N/A	N/A
Nubeqa	darolutamide	Clinical PK	Label/clinical PK	Label/clinical PK	Not mentioned	N/A	Not mentioned
Orgovyx	relugolix	N/A	Clinical PK	N/A	N/A	N/A	N/A
Orilissa	elagolix sodium	Label/clinical PK	N/A	Clinical PK	N/A	N/A	N/A
Orladeyo	berotralstat	Label/clinical PK	Clinical PK	N/A	N/A	N/A	N/A
Pemazyre	pemigatinib	Low solubility	N/A	N/A	N/A	Indirect clinical study	N/A
Pifeltro	doravirine	N/A	Low solubility	Clinical PK	N/A	N/A	N/A
Piqray	alpelisib	Clinical PK	N/A	N/A	N/A	N/A	N/A
Ponvory	ponesimod	N/A	Not mentioned	N/A	N/A	N/A	N/A
pretomanid	pretomanid	N/A	N/A	N/A	Label	N/A	N/A
Prevymis	letermovir	Clinical PK	Not mentioned	Label/clinical PK	Clinical PK	N/A	N/A
Qinlock	ripretinib	Not mentioned	Not mentioned	N/A	N/A	N/A	N/A
Retevmo	selpercatinib	PMR (clinical PK)	Not mentioned	N/A	N/A	N/A	Indirect clinical study
Reyvow	lasmiditan	Label/PMR (clinical PK)	Label/PMR (clinical PK)	N/A	N/A	N/A	N/A
Rezurock	belumosudil	PMR (clinical PK)	PMR (clinical PK)	PMR (clinical PK)	N/A	N/A	No concomitant medication
Rozlytrek	entrectinib	Clinical PK	Not mentioned	N/A	N/A	N/A	N/A
Rukobia	fostemsavir	N/A	Label/clinical PK	Label/clinical PK	N/A	N/A	N/A
Rydapt	midostaurin	Not mentioned	Not mentioned	Not mentioned	N/A	N/A	N/A
Scemblix	asciminib	Label	PMR (PBPK)	PMR (PBPK)	Not mentioned	N/A	Not mentioned
Seysara	sarecycline	Label/clinical PK	N/A	N/A	N/A	N/A	N/A
Symdeko	tezacaftor and ivacaftor	Label/clinical PK	N/A	N/A	N/A	N/A	N/A
Tabrecta	capmatinib	Label/clinical PK	Label/clinical PK	N/A	N/A	N/A	Label/indicrect clinical study
Tavalisse	fostamatinib	Label/clinical PK	Label/clinical PK	N/A	N/A	N/A	N/A
Tepmetko	tepotinib	Label/clinical PK	Indirect clinical study	N/A	N/A	N/A	N/A
Tibsovo	ivosidenib	Not mentioned	N/A	PBPK	PBPK	N/A	N/A
TPOXX	tecovirimat	Not mentioned	Not mentioned	N/A	N/A	N/A	N/A

Drug name	Active ingredient	P-gp	BCRP	OATP1B1/1B3	OAT1/3	OCT2	MATE1/2-K
Trikafta	elexacaftor, ivacaftor, and tezacaftor	Label	N/A	Label	N/A	N/A	N/A
Truseltiq	infigratinib	N/A	PMR (clinical PK)	N/A	N/A	N/A	N/A
Tukysa	tucatinib	Label/clinical PK	Not mentioned	N/A	N/A	Clinical PK	Clinical PK
Turalio	pexidartinib	Clinical PK	Not mentioned	N/A	N/A	N/A	N/A
Ukoniq	umbralisib	PMR (clinical PK)	N/A	N/A	N/A	N/A	N/A
Veklury	remdesivir	N/A	N/A	Short dosing duration	N/A	N/A	Short dosing duration
Verzenio	abemaciclib	Clinical PK	Not mentioned	N/A	N/A	Clinical PK	Clinical PK
Vizimpro	dacomitinib	Not mentioned	Not mentioned	N/A	N/A	N/A	N/A
Vosevi	sofosbuvir, velpatasvir, and voxilaprevir	Label/clinical PK	Label/clinical PK	Label/clinical PK	N/A	N/A	N/A
Vyndaqel	tafamidis meglumine	N/A	Label	N/A	Static mechanistic model	N/A	N/A
Xadago	safinamide	N/A	Label/PMR (clinical PK)	N/A	N/A	N/A	N/A
Xenleta	lefamulin	Clinical PK	Indirect clinical study	N/A	N/A	N/A	Short dosing duration
Xermelo	telotristat ethyl	Clinical PK	Indirect clinical study	N/A	N/A	N/A	N/A
Xofluza	baloxavir marboxil	Clinical PK	N/A	N/A	N/A	N/A	N/A
Xospata	gilteritinib	N/A	Not mentioned	N/A	N/A	N/A	Clinical PK
Xpovio	selinexor	N/A	N/A	Not mentioned	N/A	N/A	N/A
Zejula	niraparib	N/A	Not mentioned	N/A	N/A	N/A	N/A
Zemdri	plazomicin	N/A	N/A	N/A	N/A	N/A	Clinical PK
Zokinvy	lonafarnib	Label/clinical PK	Label/clinical PK	N/A	N/A	N/A	N/A

N/A, not available; PBPK, physiologically based pharmacokinetics; PK, pharmacokinetics; PMR, post-marketing requirement

Supplemental Table S4. Follow-up actions for *in vitro* transporter substrate drugs or rationales for no labeling

Drug name	Active ingredient	p-gp	BCRP	OATP1B1/1B3	OAT1/3	OCT2	MATE1/2-K
Aemcolo	rifamycin	Low solubility	N/A	N/A	N/A	N/A	N/A
Akynzeo	fosnetupitant and palonosetron	Not mentioned	N/A	N/A	N/A	N/A	N/A
Aliqopa	copanlisib	Not mentioned	Not mentioned	N/A	N/A	N/A	N/A
Alunbrig	brigatinib	High permeability	High permeability	N/A	N/A	N/A	N/A
Artesunate	artesunate	Not mentioned	Not mentioned	N/A	N/A	N/A	N/A
Austedo	deutetrabenazine	N/A	N/A	N/A	Not mentioned	N/A	N/A
Balversa	erdafitinib	Not mentioned	N/A	N/A	N/A	N/A	N/A
Barhemsys	amisulpride	Not mentioned	Not mentioned	N/A	N/A	N/A	Short dosing duration
Baxdela	delafloxacin	Not mentioned	Not mentioned	N/A	N/A	N/A	N/A
benznidazole	benznidazole	Not mentioned	N/A	N/A	Not mentioned	N/A	N/A
Bevyxxa	betrixaban	Label/clinical PK	N/A	N/A	N/A	N/A	N/A
Biktarvy	bictegravir, embitcitabine, and tenofovir alafenamide	Indirect clinical study	Not mentioned	N/A	N/A	N/A	N/A
Braftovi	encorafenib	Indirect clinical study	N/A	N/A	N/A	N/A	N/A
Brexafemme	ibrexafungerp	Clinical PK	N/A	N/A	N/A	N/A	N/A
Brukinsa	zanubrutinib	High permeability	N/A	N/A	N/A	N/A	N/A
Byfavo	remimazolam	Not mentioned	Not mentioned	N/A	N/A	N/A	N/A
Bylvay	odevixibat	Clinical PK	N/A	N/A	N/A	N/A	N/A
Cabenuva	cabotegravir and rilpivirine	High permeability	High permeability	Not major elimination route	Not mentioned	N/A	N/A
Calquence	acalabrutinib	Not mentioned	Not mentioned	N/A	N/A	N/A	N/A
Copiktra	duvelisib	Not mentioned	Not mentioned	N/A	N/A	N/A	N/A
Cosela	trilaciclib	IV/no safety concern	IV/no safety concern	N/A	N/A	N/A	N/A
Daurismo	glasdegib	Not mentioned	Not mentioned	N/A	N/A	N/A	N/A
Dayvigo	lemborexant	PMR (in vitro study)	N/A	N/A	N/A	N/A	N/A
Doptelet	avatrombopag	Clinical PK	N/A	N/A	N/A	N/A	N/A
Emflaza	deflazacort	Not mentioned	N/A	N/A	N/A	N/A	N/A
Enhertu	fam-trastuzumab deruxtecan-nxki	Clinical PK	Clinical PK	Clinical PK	N/A	N/A	Not mentioned
Epidioloex	cannabidiol	Not mentioned	N/A	N/A	N/A	N/A	N/A
Evrysdi	risdiplam	High permeability	High permeability	N/A	N/A	N/A	N/A
Exkivity	mobocertinib	High permeability	Not mentioned	N/A	N/A	N/A	N/A

Drug name	Active ingredient	p-gp	BCRP	OATP1B1/1B3	OAT1/3	OCT2	MATE1/2-K
Gavreto	pralsetinib	Label/PMR (clinical PK)	High permeability	N/A	N/A	N/A	N/A
Gemtesa	vibegron	Clinical PK	N/A	N/A	N/A	N/A	N/A
Ibsrela	tenapanor	Not mentioned	N/A	N/A	N/A	N/A	N/A
Idhifa	enasidenib	Not mentioned	Not mentioned	N/A	N/A	N/A	N/A
Inrebic	fedratinib	Not mentioned	N/A	N/A	N/A	N/A	N/A
Isturisa	osilodrostat	Weak substrate	N/A	N/A	N/A	N/A	N/A
Kerendia	finerenone	High permeability	N/A	N/A	N/A	N/A	N/A
Kisqali	ribociclib	Weak substrate	N/A	N/A	N/A	N/A	N/A
Koselugo	selumetinib	Not mentioned	Not mentioned	N/A	N/A	N/A	N/A
Livtencity	maribavir	Clinical PK	Wide safety range	N/A	N/A	N/A	N/A
Lumakras	sotorasib	High permeability	N/A	N/A	N/A	N/A	N/A
Lupkynis	voclosporin	Label/clinical PK	N/A	N/A	N/A	N/A	N/A
Mavyret	glecaprevir and pibrentasvir	Label/clinical PK	Label	Label/clinical PK	N/A	N/A	N/A
Mektovi	binimetinib	Not major elimination route	Not major elimination route	N/A	N/A	N/A	N/A
Motegrity	prucalopride	Clinical PK	Clinical PK	N/A	N/A	N/A	N/A
Moxidectin	moxidectin	N/A	Weak substrate	N/A	N/A	N/A	N/A
Mulpleta	lusutrombopag	Clinical PK	Clinical PK	N/A	N/A	N/A	N/A
Nerlynx	neratinib maleate	Not mentioned	N/A	N/A	N/A	N/A	N/A
Nexletol	bempedoic acid	Not mentioned	Not mentioned	N/A	Not mentioned	N/A	N/A
Nextstellis	drospirenone and estetrol tablets	High permeability	High permeability	N/A	N/A	N/A	N/A
Nubeqa	darolutamide	Label/clinical PK	Not mentioned	N/A	N/A	N/A	N/A
Nulibry	fosdenopterin	N/A	N/A	N/A	N/A	N/A	Weak substrate
Nurtec ODT	rimegepant	Label/PMR (clinical PK)	Label/PMR (clinical PK)	N/A	N/A	N/A	N/A
Nuzyra	omadacycline	Clinical PK	N/A	N/A	N/A	N/A	N/A
Olinvyk	oliceridine	Not mentioned	N/A	N/A	N/A	N/A	N/A
Olumiant	baricitinib	Clinical PK	Clinical PK	N/A	Label/clinical PK	N/A	indirect clinical study
Ongentys	opicapone	Clinical PK	Not mentioned	Not mentioned	N/A	N/A	N/A
Orgovyx	relugolix	Label/PMR (clinical PK)	N/A	N/A	N/A	N/A	N/A
Orilissa	elagolix sodium	Not mentioned	N/A	Label/clinical PK	N/A	N/A	N/A
Orladeyo	berotralstat	Label/clinical PK	Label/clinical PK	N/A	N/A	N/A	N/A
Pemazyre	pemigatinib	saturation	saturation	N/A	N/A	N/A	N/A
Pifeltro	doravirine	High permeability	N/A	N/A	N/A	N/A	N/A
Piqray	alpelisib	Weak substrate	Label	N/A	N/A	N/A	N/A
Prevymis	letermovir	High permeability	N/A	Label/clinical PK	N/A	N/A	N/A

Drug name	Active ingredient	p-gp	BCRP	OATP1B1/1B3	OAT1/3	OCT2	MATE1/2-K
Qinlock	ripretinib	Not mentioned	Not mentioned	N/A	N/A	N/A	N/A
Qulipta	atogepant	Clinical PK	PBPK	Label/clinical PK	Not major elimination route	N/A	N/A
Radicava	edaravone	N/A	Not mentioned	N/A	Weak substrate	N/A	N/A
Recarbrio	imipenem, cilastatin, and relebactam	N/A	N/A	N/A	Clinical PK	N/A	Not mentioned
Retevmo	selpercatinib	Clinical PK	Not mentioned	N/A	N/A	N/A	N/A
Reyvow	lasmiditan	High permeability	N/A	N/A	N/A	N/A	N/A
Rezurock	belumosudil	Not mentioned	N/A	Not major elimination route	Not major elimination route	N/A	Not major elimination route
Rinvoq	upadacitinib	PBPK	PBPK	N/A	N/A	N/A	N/A
Rozlytrek	entrectinib	Not mentioned	Not mentioned	N/A	N/A	N/A	N/A
Rukobia	fostemsavir	High permeability	High permeability	N/A	N/A	N/A	N/A
Scemblix	asciminib	Clinical PK	Not mentioned	N/A	N/A	N/A	N/A
Steglatro	ertugliflozin	Weak substrate	Weak substrate	N/A	N/A	N/A	N/A
Sunosi	solriamfetol	N/A	N/A	N/A	N/A	Weak substrate	Weak substrate
Symdeko	tezacaftor and ivacaftor	Clinical PK	Not mentioned	Not mentioned	N/A	N/A	N/A
Symproic	naldemedine	Label/clinical PK	N/A	N/A	N/A	N/A	N/A
Tabrecta	capmatinib	High permeability	N/A	N/A	N/A	N/A	N/A
Talzenna	talazoparib	Label/PMR (clinical PK)	Label	N/A	N/A	N/A	N/A
Tavalisse	fostamatinib	Not mentioned	N/A	N/A	N/A	N/A	N/A
Tavneos	avacopan	Not mentioned	N/A	N/A	N/A	N/A	N/A
Tazverik	tazemetostat	saturation	N/A	N/A	N/A	N/A	N/A
Tepmetko	tepotinib	Label/clinical PK	N/A	N/A	N/A	N/A	N/A
Tibsovo	ivosidenib	Not mentioned	N/A	N/A	N/A	N/A	N/A
Trikafta	elexacaftor, ivacaftor, and tezacaftor	Not mentioned	Not mentioned	Not mentioned	N/A	N/A	N/A
Truseltiq	infigratinib	PMR (clinical PK)	PMR (clinical PK)	N/A	N/A	N/A	N/A
Tukysa	tucatinib	High permeability	High permeability	N/A	N/A	N/A	N/A
Ubrelvy	ubrogepant	Label	Label	Weak substrate	Weak substrate	N/A	N/A
Veklury	remdesivir	PMR (clinical PK)	N/A	PMR (clinical PK)	N/A	N/A	N/A
Verquvo	vericiguat	Clinical PK	Not mentioned	N/A	N/A	N/A	N/A
Verzenio	abemaciclib	Not mentioned	Not mentioned	N/A	N/A	N/A	N/A
Vitrakvi	larotrectinib	Clinical PK	Clinical PK	N/A	N/A	N/A	N/A
Vizimpro	dacomitinib	Not mentioned	Not mentioned	N/A	N/A	N/A	N/A
Vosevi	sofosbuvir, velpatasvir, and voxilaprevir	Label	Label	Label/clinical PK	N/A	N/A	N/A
Welireg	belzutifan	Weak substrate	N/A	Weak substrate	N/A	N/A	N/A
Xenleta	lefamulin	label	N/A	N/A	N/A	N/A	N/A

Drug name	Active ingredient	p-gp	BCRP	OATP1B1/1B3	OAT1/3	OCT2	MATE1/2-K
Xerava	eravacycline	Weak substrate	Weak substrate	N/A	N/A	N/A	N/A
Xermelo	telotristat ethyl	Not mentioned	N/A	N/A	N/A	N/A	N/A
Xofluza	baloxavir marboxil	Clinical PK	N/A	N/A	N/A	N/A	N/A
Xospata	gilteritinib	Label	N/A	N/A	N/A	N/A	N/A
Yupelri	revefenacin	Wide safety range	Wide safety range	Label	N/A	N/A	N/A
Zejula	niraparib	Not mentioned	Not mentioned	N/A	N/A	N/A	N/A
Zeposia	ozanimod	Clinical PK	Label/clinical PK	N/A	N/A	N/A	N/A
Zepzelca	lurbinectedin	Not mentioned	N/A	N/A	N/A	N/A	N/A
Zokinvy	lonafarnib	Weak substrate	N/A	N/A	N/A	N/A	N/A

N/A, not available; PBPK, physiologically based pharmacokinetics; PK, pharmacokinetics; PMR, post-marketing requirement
